# Occurrence of *Porphyromonas gingivalis* fimA type II and prtC genotype among periodontitis patients

**DOI:** 10.1186/1471-2334-12-S1-P22

**Published:** 2012-05-04

**Authors:** Mahalakshmi Krishnan, Padma Krishnan, Subashini Natarajan

**Affiliations:** 1 Department of Microbiology, Dr. ALM PGIBMS, University of Madras, Chennai, India; 2 Department of Microbiology, Sree Balaji Dental College and Hospital, Chennai, India; 3 Department of Periodontics and Implantology, Tamilnadu Government Dental College and Hospital, Chennai, India

## Background

*Porphyromonas gingivalis* fimbriae are classified into six genotypes (types I-V and Ib). Among them, occurrence of *fimA* type II genotype is more predominant in periodontitis patients. Similarly collagenase encoded by *prtC* gene is a potential virulence factor expressed by *P. gingivalis* strains associated with periodontal disease. The study was opted to detect the presence *of P. gingivalis fimA* type II and *prtC* genotypes in periodontitis patients.

## Methods

Subgingival plaque samples collected from 128 chronic periodontitis (ChP) and 72 aggressive periodontitis (AgP) patients were subjected to PCR to screen for the presence of *fimA* type II and *prtC* gene of *P. gingivalis.* Chi-square test was employed to compare the prevalence of the genotypes.

## Results

The prevalence of *P. gingivalis fimA* type II genotype among ChP, AgP and health was 50.5%, 45.3 % and 13.60%, respectively. While, prevalence of *P. gingivalis* prtC genotype among ChP, AgP and health was 49.5%, 45.3% and 9.10% respectively. *P. gingivalis* type II *fimA*+/*prtC* + genotype were present in 28.9% of ChP, 33.3% of AgP patients and 4.5% of healthy subjects. Patients positive for both the genes showed probing depth of ≥7mm. Significant difference was observed between periodontitis and healthy subjects for all the three genotypes (*P*=0.001).

## Conclusion

The results show that *P. gingivalis fimA* type II and *prtC* genotypes are equally associated with chronic and aggressive periodontitis. The predominance of *P. gingivalis fimA* type II+ / *prtC*+ genotype in teeth with deep pockets or serious attachment loss, suggest their role in periodontal destruction.

